# An investigation of what protective individual- and community-level factors are associated with life satisfaction in middle-aged and older family carers in Ireland

**DOI:** 10.3389/fpubh.2023.1207523

**Published:** 2023-08-10

**Authors:** Christine A. McGarrigle, Mark Ward, Rose Anne Kenny

**Affiliations:** ^1^The Irish Longitudinal Study on Ageing (TILDA), School of Medicine, Trinity College Dublin, The University of Dublin, Dublin, Ireland; ^2^St James's Hospital, Mercer's Institute for Successful Ageing, Dublin, Ireland

**Keywords:** caring, resilience, wellbeing, life satisfaction, aging

## Abstract

**Background:**

Family care plays an essential role in providing care in society. However, caring can cause stress, and mental and physical responses to caring vary widely. Different outcomes for carers may reflect different approaches or adaptability to caring and their ability to maintain or recover their mental health and wellbeing following an adverse event (psychosocial resilience). We aim to identify factors that may promote psychosocial resilience, conceptualized as maintaining or recovering subjective wellbeing and operationalized as satisfaction with life, among carers.

**Methods:**

Data were from 6 Waves (2009–2021) of The Irish Longitudinal Study on Aging (TILDA), a prospective biennial nationally representative longitudinal study of older adults aged ≥50 in Ireland. Family caregiving was assessed in Waves 3–6. Participants were asked if they cared for someone, their relationship to the recipient, and the number of hours per week that they provided care. We used growth mixture modeling to identify latent trajectories of satisfaction with life (SWL) before and after caring was initiated. Regression modeling was then used to identify protective factors (at the individual, family, and community levels) associated with resilient trajectories.

**Results:**

Overall, 731 (12.2%) participants became carers during follow-up. We identified three trajectories in SWL in carers following initiation of caring, namely, Resilient-Stable (81%), Resilient-Recovery (12%), and Non-recovery (6%). Membership in Resilient-Stable and Resilient-Recovery trajectories was associated with fewer depressive symptoms (OR = 0.86, 95% CI 0.78, 0.94) and chronic conditions (OR = 0.21, 95% CI 0.06, 0.74), larger social networks (OR = 2.03, 95% CI 1.06, 3.86), more close friends and relatives (OR = 1.15, 95% CI 1.01, 1.32), and caring for someone other than a child (OR = 0.19, 95% CI 0.07, 0.51) compared to the Non-recovery group.

**Conclusion:**

Becoming a family carer was associated with a decline in SWL over time in some carers. However, most carers either did not experience a decline in SWL or recovered their SWL over time. We found that both individual and community-level supports may be protective for carers' wellbeing. These results will inform the priorities for social and community-level services and support for older carers and contribute to the design of new projects and programs to meet these needs.

## 1. Introduction

Community-based care can facilitate aging in place, has the potential to delay admission to a nursing home for older adults, and improves quality of life (1). The provision of formal, state-provided services enables the older population to remain at home, even with increasing disability, and can work well in tandem with informal, family-provided care (1). Formal social care in Ireland is financed via the Health Service Executive (HSE) and delivered through a network of local health offices. Eligibility for state-provided care is determined by means testing and is based on individual needs, taking into account limited financial resources. This support encompasses various forms of support tailored to specific requirements. It includes home-help, where someone assists with household tasks; a personal care assistant who assists with bathing, showering, or bodily care, and respite care that involves temporary stays at external residential or day care facilities. However, in practice, most caregiving is informal, unpaid care provided by a family member. Informal care for family members, spouse, parents, disabled children, and friends is provided mainly by middle-aged and older carers, the majority of whom are women (2). Household composition, family, and social network, in addition to social policy within countries, are important determinants of receipt of both the informal and formal care (3, 4). These same determinants, along with personal beliefs and cultural factors, can all play a role in determining who becomes a carer (4). However, with continued pressures on formal state-provided services due to population aging, there may be growing demands on families, potentially leading to older family members taking on caregiving responsibilities.

Informal caregiving is associated with increased stress, depression, and ill health (5–9). A higher caregiver burden is associated with a poorer health profile and lower quality of life (8), social isolation (10), and premature mortality (11). However, not all carers experience negative health consequences, and research has identified a variety of positive gains from providing care, including a sense of reward (12), purpose, increased gratification, and the development of a closer relationship with the person receiving care (13). When perceived as a positive productive activity in later life, caring is also associated with better wellbeing (14) and a reduced risk of mortality among carers (15). Positive characteristics of the caregiver have been found to mediate the relationships between caregiving and psychological outcomes (16).

It is critical that carers maintain their own social participation and engagement alongside their caregiving roles. The importance of participation and social engagement for the health and wellbeing of older people has been well established (17, 18). Social integration has positive health effects, including reduced mortality risk for those with stronger personal relationships (19). Furthermore, social participation in leisure activities and religious activities has been associated with a lower risk of negative mental and physical health outcomes (20–22) as well as mortality risk (17). Lack of formal state-provided services to support family carers may negatively impact their ability to continue to maintain important social networks and support, leading to increased isolation and loneliness (10).

The World Health Organization's Healthy Aging model places resilience at its core, defining it as “the ability to maintain or improve a level of functional ability in the face of adversity (either through resistance, recovery or adaption)” [(23), p. 29]. Resilience has been further characterized in older age to represent both lifespan and individual and structural support systems, and these theories have been further developed with respect to the COVID-19 pandemic, incorporating the loss of social and structural supports (24–26). Some of these differences in mental health and wellbeing observed between carers may indicate how individuals vary in their ability to adapt to changing circumstances and either maintain or recover their psychosocial resilience. This means that the interplay between behavioral and social factors may help individuals emotionally adapt to adverse events, including the potential stress associated with being a family carer. This resilience in the face of adversity may explain why some carers are able to maintain their wellbeing, including their life satisfaction (27). As societies age and prioritizing resources becomes increasingly important, there is increasing interest in how psychosocial resilience can be sustained or even enhanced in old age.

The impetus for this was further enhanced by the COVID-19 pandemic, which highlighted inequities in access to healthcare and the disproportionate impact of the virus on vulnerable communities such as the older population. When behavioral and social supports were withdrawn and stay-at-home orders were in place, the proportion of formal to informal care changed substantially with informal caregiving by older people in Ireland increasing 3-fold during this period (6). Similar increases in both caregiver burden and caregiving intensity were also described in the United States (28), Canada (29), and other European countries (30).

Resilience is viewed as a modifiable characteristic that can be strengthened over time through a developmental process (27). Higher life satisfaction has been associated with better subsequent physical and psychological health outcomes in older adults, indicating policies aimed at enhancing several indicators of psychosocial wellbeing, health behaviors, and physical outcomes in older people could focus on greater life satisfaction as an outcome (31). Notably, life satisfaction has been found to be more responsive to change following adverse events compared to positive affect (32). Additionally, the associations between social exchanges and life satisfaction become stronger with advancing older age, particularly when functional decline and increasing reliance on social networks occur (32). Considering resilience as the adaptation or recovery following adverse events in older age, trends in satisfaction with life serve as suitable indicators of resilience. These trends align with the theory of psychosocial resilience in older age, particularly in relation to chronic disease accumulation (26). Although life satisfaction is generally believed to remain relatively stable despite age-related losses, this is not consistent for all individuals (33).

In the context of an aging population, there are growing concerns about the sustainability of government expenditures on healthcare and social services. To aid policymakers in making informed decisions, a robust evidence base is required. Using data from a nationally representative longitudinal study of older adults, this study describes differences in the wellbeing of carers and identifies factors at the individual- and community-level that explain these differences. Specifically, this study focuses on the psychological wellbeing of older carers and examines protective factors that contribute to the maintenance, or adaptation, and recovery of wellbeing following the commencement of caregiving. Satisfaction with life is used as a measure of subjective wellbeing. By examining the different patterns of life satisfaction over time, this study will inform the priorities for social and community-level services and supports for carers and contribute to the design of new projects and programs to help inform policy change.

## 2. Materials and methods

### 2.1. Study sample

This study includes participants aged ≥50 years from the Irish Longitudinal Study on Aging (TILDA), a nationally representative study of community-dwelling adults in the Republic of Ireland. The analytic sample included participants who took part in at least three waves of data collection, at least one before caring, one when caring commenced, and one after caring commenced, between Wave 1 (2009–2011) and Wave 6 (2020–2021) (average follow-up period of 10 years). Details of the cohort and study procedure are described elsewhere (34). Briefly, at TILDA Wave 1, 8, 171 adults aged ≥50 years completed a computer-assisted personal interview (CAPI) in their own home, and 85% (*n* = 6, 911) of these also completed a self-completion questionnaire (SCQ). Data were then collected from these same participants every 2 years. Wave 6 data were collected via computer-assisted telephone interview (CATI) due to COVID-19 pandemic restrictions. Response rates for TILDA for each wave were as follows: Wave 2 (88%), Wave 3 (85%), Wave 4 (84%), Wave 5 (81%), and Wave 6 (85%). Ethical approval for TILDA was received from the Research Ethics Committee of the Faculty of Health Sciences, Trinity College Dublin.

### 2.2. Measures

#### 2.2.1. Caring measures

Questions about caring were first collected in TILDA at Wave 3 (2014), and we included in our analyses all individuals who transitioned into and out of caring between Wave 3 and Wave 6 (*n* = 731).

From Wave 3 (2014) onwards, participants were asked the following question: “Did you care for someone in the past week, by care for someone, we mean the active provision of care.” If they did care for someone, they were then asked about their relationship to the care recipient (spouse, children, other relative, friend, or neighbor) and the number of hours per week that they provided care. Children included adopted and stepchildren (age range 8–56, Wave 3). With this information, we created a caring intensity variable coded 0–1: low to moderate intensity caregiving (1–49 h of caregiving in the last week), and high intensity caregiving (≥50 h in the last week). Data from Wave 3 (2014), Wave 4 (2016), Wave 5 (2018), and Wave 6 (2020–2021) were used to capture transitions into and out of caring. Participants were grouped as having “never cared,” “currently caring,” and “past carer.”

#### 2.2.2. Outcome measures

Self-rated life satisfaction was used to indicate psychosocial resilience. Satisfaction with life assesses an individual's judgment of his or her life by using their own criteria, is a global assessment of how they evaluate their life, not their current feelings, and is a relatively stable phenomenon (35).

*Self-rated life satisfaction* (SWL) was measured using a single-item question. The participant was asked to rate how satisfied they are with their life from 1 to 10, with higher scores indicating better SWL (1 = Not at all satisfied, 10 = Completely satisfied).

#### 2.2.3. Protective factors

Potentially associated variables included the following sociodemographic characteristics: age (continuous), sex, highest educational attainment (primary, 8 years; secondary, 13 years; or tertiary, >13 years), and marital status (never married, married, separated/divorced, and widowed). Health variables included a count of chronic conditions (heart attack/heart failure/angina, cataracts, hypertension, high cholesterol, stroke, diabetes, lung disease, asthma, arthritis, osteoporosis, cancer, Parkinson's disease, peptic ulcer, and hip fracture) and the presence of both difficulties with activities of daily living (ADL) and instrumental activities of daily living (IADL). The list of ADLs included if participants had difficulties walking across a room, dressing, bathing, eating, getting in and out of bed, and using the toilet. IADLs included if participants had difficulties preparing meals, shopping for groceries, making telephone calls, taking medications, and managing money, such as paying bills and keeping track of expenses. These were grouped as not disabled, IADL disability only, or any ADL disability. Baseline depressive symptoms were assessed using the short, 8-item version of the Center for Epidemiological Studies Depression (CES-D) scale. Scores range from 0 to 24 with higher scores indicate a greater frequency of depressive experiences (36).

*Social network* was measured using the Berkman-Syme Social Network Index (17). This is a composite scale scored 0–4 that captures four types of social connection, namely, married, number of close ties with friends, family and children, member of a church, and member of voluntary organizations including clubs. A score of 0–1 indicates a participant who is most isolated, while a score of 4 indicates that they are most integrated. We also included a list of close relatives and friends. Church attendance in Ireland is an important source of social support and integration for the older generation, and overall, 60% report attending church services at least once a week (21).

#### 2.2.4. State-provided formal care

Participants were asked if they received state-provided personal care attendants (a person employed by the state to assist with bathing and bodily care) or home help (a person employed by the state to help with household chores). This was operationalized as “No receipt” or “Received in the past year.”

### 2.3. Statistical methods

The first time point at which care was reported was considered anchor time 0. Latent SWL trajectories were then derived from the three time points preceding time zero and the three following the inception of caregiving. This captures a total of six waves (3 before and 3 after). Participants were included if they took part in at least three study waves and had measurements before and after the wave at which they started caring. We used growth mixture modeling (GMM) techniques using a censored normal distribution to identify distinct latent clusters of trajectories (groups) of individuals' levels of SWL between Wave 1 and Wave 6 using the Stata traj plugin (37). First, the number of groups that best described the data was chosen based on the model with the best fit indices. Models were evaluated using the relative fit information criteria of Bayesian Information Criteria (BIC). Lower BIC are preferred, and a reduction of >10 between models is considered to indicate a better fit. Second, we considered entropy and posterior class membership. Entropy is a summary measure of the uncertainty of the classification of individuals into trajectory groups (range 0–1). An entropy >0.8 indicates that individuals are likely to be in that class and that there is adequate separation between classes. We favored models with higher entropy when selecting among models with similar fit indices. Finally, we considered the size and interpretability of classes and selected only models with values >5.0 for the odds of correct classification based on the weighted posterior probabilities, average posterior probability per group >0.7, and total probability based on posterior probabilities >0.05. We defined a resilient trajectory as one that showed either a deterioration in SWL at the beginning of care, which was then followed by a “recovery” indicated by an improvement in SWL, or a robust trajectory, where there was no indication of a decline. Once the trajectories were identified, logistic regression was used to estimate the likelihood of membership in the resilient trajectory class based on sociodemographic, health, caring type, formal and social support characteristics.

As a sensitivity analysis, we selected a randomized sample of age and sex 1:1 matched controls who were not carers using the stata command ccmatch. Similar to the main SWL trajectory analysis, each control had to have at least one point prior to and post a nominal t0 matched to the case. GMM was used to model and identify clusters of SWL in controls, similar to what was described for carers.

In addition, we evaluated whether associations between caring and SWL were similar in men and women by including an interaction term for gender in the logistic regression analysis (6).

## 3. Results

**Figure 1:**
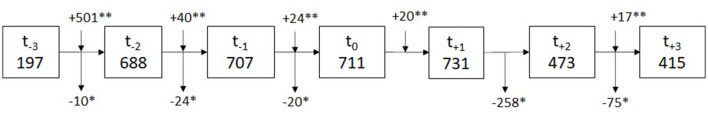
Participation of carers in study waves before and after caring commenced. *Did not take part in this wave of data collection; **rejoined for the subsequent wave. *t*_0_ is time participant became a carer, *t*_−1_ is the interview prior to *t*_0_ (−2 years) and *t*_+1_ is the interview after becoming a carer (+2 years).

Figure 1 shows the participation of carers through data collection waves both before and after caring commenced. The characteristics of the carers in the sample at baseline are shown in Table 1. More carers were women (67%), the mean age of carers was 64.31 years, and the majority was married (76.9%) and 9.2% widowed. One-third of carers were in employment, while a low proportion had a functional disability (2% and 4% had IADL and ADL difficulty, respectively). Most care was provided to another relative (30.4%), while 18.6% reported caring for their spouse and 8.8% for their child.

**Table 1:** Descriptive statistics of carers in the TILDA sample at Wave 3 (2014).

	**Wave 3 (*n =* 731)**
	**Mean (SD)/*****n*** **(%)**
Age	64.31 (7.3)
Sex [*n* (%) Male]	235 (33.3)
**Educational attainment [*****n*** **(%)]**	
Primary	125 (8.0)
Secondary	269 (38.7)
Higher	302 (43.4)
**Marital status [*****n*** **(%)]**	
Married	535 (76.9)
Single	40 (5.8)
Separated/Divorced	57 (8.3)
Widowed	64 (9.2)
**Employment status [*****n*** **(%)]**	
Employed	275 (36.6)
Retired	286 (38.0)
Other	191 (25.4)
Not disabled [*n* (%)]	681 (94.2)
IADL disability only [*n* (%)]	14 (2.0)
Any ADL disability [*n* (%)]	29 (4.2)
Partner has ADL or IADL disability [*n* (%)]	67 (9.5)
**Count of chronic conditions [*****n*** **(%)]**	**0.96 (0.9)**
0	268 (37.1)
1	273 (37.8)
2	137 (19.0)
≥3	45 (6.3)
Baseline depressive symptoms (CES-D8)	2.80 (3.5)
**Recipient of care [*****n*** **(%)]**	
Spouse	130 (18.6)
Child	62 (8.8)
Grandchild	78 (11.1)
Other relative	214 (30.4)
Friend or neighbor	105 (14.9)
Years of caring	2.4 (0.9)
High hours of caring (50+/week) [*n* (%)]	108 (15.3)
**Formal support [*****n*** **(%)]**	
Carer received home help	11 (1.6)
Partner received home help	19 (2.7)
**Social support**	
Social integration score	3.0 (0.9)
Number of close relatives or friends	10.1 (5.3)
**Satisfaction with life score**	
*t*_−2_ before starting caring	6.23 (1.0)
*t*_−1_ before starting caring	6.16 (1.1)
*t*_0_ starting caring	6.12 (1.2)
*t*_+1_ after starting caring	6.09 (1.0)
*t*_+2_ after starting caring	6.05 (1.0)

SD, standard deviation; IADL, instrumental activities of daily living; ADL, activities of daily living; CES-D8, Center for Epidemiological Studies Depression scale.

Social integration score is measured through the Berkman-Syme index (range 0–4); t_0_ is time participant became a carer, *t*_−1_ is the interview prior to t_0_ (−2 years), and t_+1_ is the interview after becoming a carer (+2 years).

Chronic conditions include heart attack/heart failure/angina, cataracts, hypertension, high cholesterol, stroke, diabetes, lung disease, asthma, arthritis, osteoporosis, cancer, Parkinson's disease, peptic ulcer, and hip fracture.

### 3.1. Latent class trajectories of mental health and wellbeing around caring initiation

**Figure 2:**
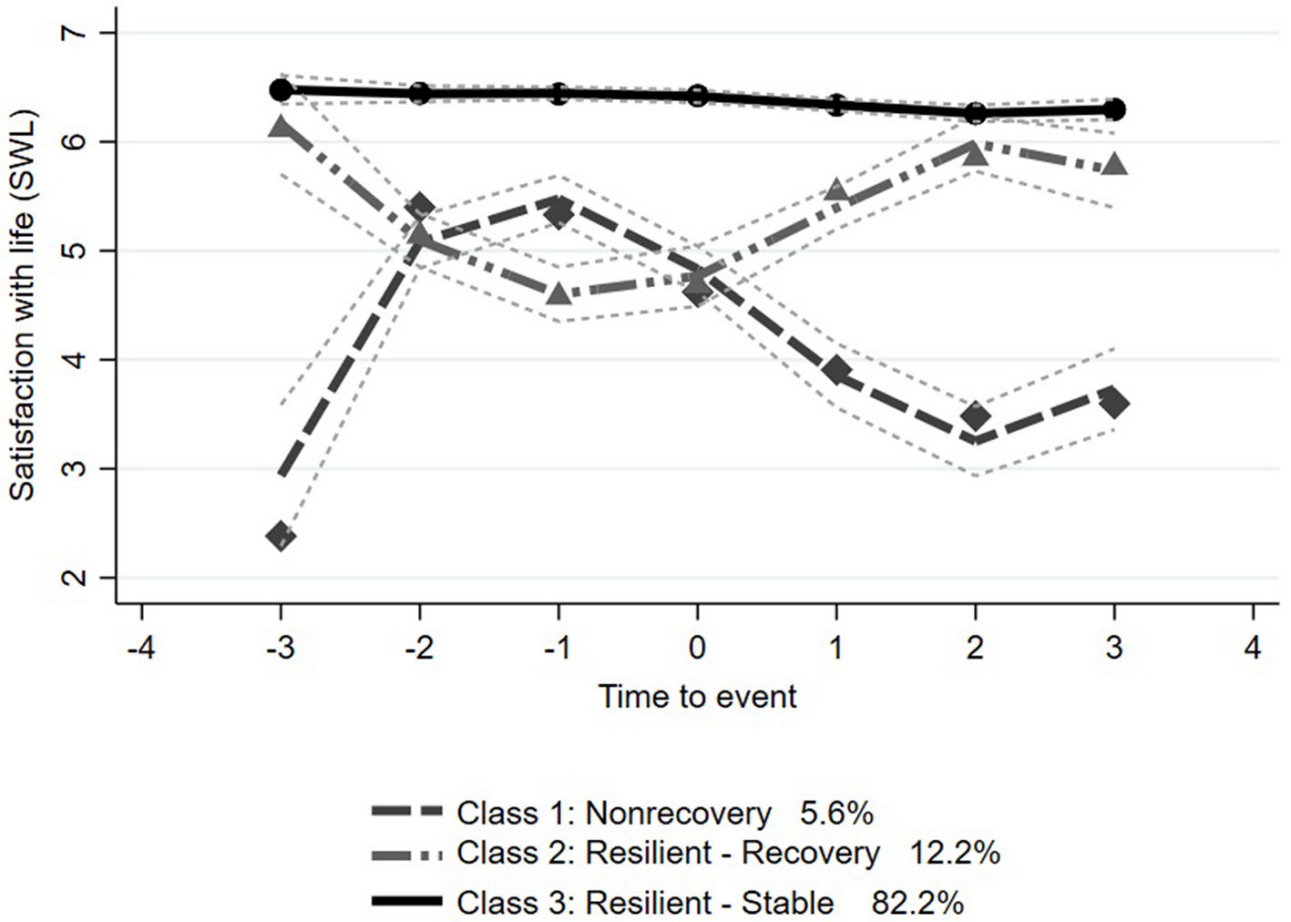
Latent growth trajectories (with 95% confidence interval) of satisfaction with life by time from becoming a carer (*t*_0_): Class 1 = Nonrecovery, Class 2 = Resilient-Recovery, and Class 3 = Resilient-Stable. *t*_0_ is time participant became a carer, t_−1_ is the interview prior to *t*_0_ (−2 years), and t_+1_ is the interview after becoming a carer (+2 years).

Supplementary Table 1 presents the final fit statistics for the latent class group analysis. Posterior probabilities, odds of correct classification, and entropy were high, indicating that individuals were likely to be in that class and that there was adequate separation between classes. The latent class trajectories of carers' SWL before and after they became carers, denoted as time 0, are shown in Figure 2. The solid lines represent the parameter estimates of the model, and the dashed lines form the 95% confidence interval on the estimated probabilities of resilient class membership. The point estimates were calculated using each individual's responses weighted based on posterior probabilities of class membership (37).

The best fit statistics identified three trajectory classes in SWL. In the Resilient-Stable trajectory, estimated to include 82.2% (*n* = 606) of the sample of carers, carers started with high SWL and remained stable both before and after becoming carers. In the Nonrecovery trajectory, including 12.2% (*n* = 83) of the sample, carers started with a high SWL score and declined before becoming a carer and then recovered 4 years after t0. In the Nonrecovery trajectory, including 5.6% (*n* = 42) of the sample, carers started with a lower SWL score and declined rapidly following becoming carers at t0. SWL scores remained above 6 in the Resilient-Stable class, both before and after the initiation of caring. SWL score in the Resilient-Recovery class started around 6, dropped to below 5 when caring started, but started to recover by the next study wave and had returned to pre-caring score by the second study wave after becoming a carer. The mean SWL score in the Nonrecovery trajectory decreased after the initiation of caring and had not recovered three study waves later (6 years), remaining below 4.

When compared to SWL trajectories for age- and sex-matched non-carers (Supplementary Figure 1), two trajectory classes in SWL were identified. One Stable-High (including 89.0% (*n* = 650) of the sample) where non-carers' SWL remained stable over follow-up, and a second Stable-Low (including 11% (*n* = 81) of the sample) which started low and remained stable over follow-up.

### 3.2. Independent variables associated with trajectory membership

Resilient-Stable and Resilient-Recovery classes were combined as resilient and compared to Nonrecovery class membership. Table 2 shows the relationship between sociodemographic, health, caring, and formal and informal support characteristics associated with membership in an SWL resilient trajectory class. The results show that carers with better physical and mental health, lower depressive symptoms, and a lower number of chronic conditions had a higher probability of being in the resilient SWL trajectories. Caring for someone other than a child and caring for a shorter duration were also predictors of resilient class membership. Social support, measured by both higher social integration and a higher number of close friends and relatives, was associated with resilient trajectory membership. The likelihood of resilient group membership increased 2-fold per point increase on the social integration scale and by 15% per increasing number of close friends or relatives. There was no evidence that the receipt of formal state-provided support was associated with resilience class membership. Results were similar for both men and women.

**Table 2:** Relationship of sociodemographic, health, formal, and social support characteristics at baseline with probability of resilient satisfaction with life (OR) class membership based on incident caring.

	**Model 1**	**Model 2**	**Model 3**
	**OR [95% CI]**	**OR [95% CI]**	**OR [95% CI]**
Age	1.01 [0.96, 1.07]	1.00 [0.94, 1.07]	0.99 [0.92, 1.06]
Sex (base men)	1.14 [0.57, 2.35]	1.13 [0.53, 2.42]	0.89 [0.40, 1.98]
**Education (base primary)**			
Secondary	0.40 [0.13, 1.27]	0.35 [0.10, 1.21]	0.23^*^ [0.06, 0.94]
Tertiary	0.49 [0.15, 1.59]	0.39 [0.11, 1.38]	0.21^*^ [0.05, 0.87]
**Employment status (base employed)**			
Retired	0.57 [0.22, 1.45]	0.93 [0.35, 2.48]	1.00 [0.36, 2.79]
Other	0.65 [0.28, 1.55]	1.10 [0.43, 2.83]	1.22 [0.46, 3.25]
**Marital status (base married)**			
Single	0.65 [0.14, 3.06]	0.67 [0.13, 3.45]	1.58 [0.24, 10.18]
Separated/divorced	0.29^*^ [0.11, 0.73]	0.38 [0.13, 1.05]	0.93 [0.25, 3.40]
Widowed	0.76 [0.20, 2.86]	0.82 [0.21, 3.22]	1.16 [0.26, 5.30]
**Caring**			
**Care recipient**			
Spouse	0.56 [0.23, 1.34]	0.73 [0.28, 1.90]	0.89 [0.31, 2.56]
Child	0.22^***^ [0.09, 0.51]	0.22^**^ [0.09, 0.55]	0.19^***^ [0.07, 0.51]
Grandchild	1.61 [0.45, 5.78]	1.81 [0.49, 6.70]	1.85 [0.48, 7.18]
Other relative	0.52 [0.26, 1.06]	0.51 [0.24, 1.08]	0.47 [0.21, 1.05]
Friend	1.10 [0.40, 3.06]	0.76 [0.26, 2.20]	0.66 [0.22, 1.98]
Care ≥50 h/week	0.86 [0.33, 2.25]	0.96 [0.35, 2.61]	1.21 [0.42, 3.52]
Increasing years of caring	0.85 [0.63, 1.16]	0.85 [0.62, 1.17]	0.77 [0.56, 1.07]
**Health**			
Carer IADL only		0.40 [0.09, 1.32]	0.52 [0.09, 1.95]
Carer any ADL		0.35 [0.09, 1.32]	0.44 [0.10, 1.89]
Spouse any disability		0.69 [0.25, 1.85]	0.59 [0.20, 1.79]
**Number of chronic conditions (base 0)**			
1		0.40^*^ [0.16, 0.99]	0.34^*^ [0.13, 0.87]
2		0.76 [0.21, 2.67]	0.75 [0.19, 2.99]
≥3		0.22^*^ [0.06, 0.74]	0.21^*^ [0.06, 0.74]
Baseline CES-D8 score		0.87^***^ [0.80, 0.94]	0.86^**^ [0.78, 0.94]
**Social support**			
Social integration			2.03^*^ [1.06, 3.86]
Number of close friends and relatives			1.15^*^ [1.01, 1.32]
**State-provided formal support**			
Formal support received by carer			0.82 [0.18, 3.73]
Formal support received by partner			0.64 [0.11, 3.75]

Panel shows the odds ratio (OR), 95% confidence intervals (CI) (Model 1-3), of being in Resilient-Recovery and Resilient-Stable satisfaction with life trajectory over time since becoming a carer. Model 1 adjusts for sociodemographic characteristics and type of care; Model 2 adjusts for Model 1 + health; Model 3 adjusts for Model 2 + formal support + social support variables.

^*^p < 0.05, ^**^p < 0.01, ^***^p < 0.001.

SD, standard deviation; IADL, Instrumental Activities of Daily Living; ADL, Activities of Daily Living; CES-D8, Center for Epidemiological Studies Depression scale.

Social integration score is measured through the Berkman-Syme index (range 0–4); Chronic conditions include heart attack/heart failure/angina, cataracts, hypertension, high cholesterol, stroke, diabetes, lung disease, asthma, arthritis, osteoporosis, cancer, Parkinson's disease, peptic ulcer, and hip fracture.

## 4. Discussion

Our study adds to the existing body of knowledge on caregiving by providing compelling evidence of high levels of resilience in subjective wellbeing in older carers among a nationally representative cohort of older adults. By examining the trajectories of carers' life satisfaction before and after they assumed their caregiving role, we showed that while a small proportion of carers never recovered their wellbeing following the initiation of caregiving, the majority either had no change in subjective wellbeing or recovered their wellbeing to pre-caregiving level within 4 years.

We demonstrated that strong social support was important for resilience among carers. Carers who were more socially integrated into their community were twice as likely to be on a resilient trajectory. Similarly, a larger number of close relatives and friends also had a protective effect. These findings are similar to those reported in studies of resilience in carers of people living with dementia that also found that higher family support (38) and network size and satisfaction with social support were associated with resilience (39). In addition to support and community engagement, previous research has shown that reward and gratitude are important for overall life satisfaction among caregivers (14). The protective outcomes observed are likely due to the beneficial influence of communication and social support, which contribute to the fostering of resilient coping mechanisms (40). Reviews of studies on resilience in the general population during the COVID-19 pandemic suggest that regulatory flexibility and positive appraisal styles are likely mechanisms for promoting resilience (41). These, in turn, were found to play a mediating role in the relationship between social support and resilient outcomes in cross sectional analyses (42, 43). This concurs with previous research that has established that additional social interaction and supportive family and social circles, including religious and community resources, can offset adverse effects on health and wellbeing (21, 22, 32, 44, 45). Similarly, the negative effect of the COVID-19 pandemic on the mental health and wellbeing of carers has demonstrated the importance of social supports (6, 29). Interventions to promote social participation of the older population through volunteering, such as Experience Corps (46) or community centers (47) have demonstrated successful increases in participation among the older population taking part (48) and subsequent improvements in self-rated mental and physical health (47, 49). Consideration should be given to interventions aimed at enhancing the social integration of carers as volunteering may not always be a suitable option for carers who are already overwhelmed with their caregiving responsibilities.

Receipt of formal state-provided support was not associated with resilience in this study. Similarly, a study conducted in Japan found that although informal social support was associated with lower caregiver burden, formal social support had no such association (50). The authors concluded that while formal care provides instrumental assistance, it does not provide emotional support, which has been argued to have a more direct and positive influence on psychological wellbeing, particularly when provided by close family members. However, in this study we only measure formal support received within the home, and this does not include support received by care recipients living outside of the home, so we may have underestimated the amount of formal support that may have been received by care recipients.

In terms of health-related indicators, we found that baseline depressive symptoms and multiple chronic conditions were associated with subsequent membership in the Nonrecovery trajectory in SWL. Depressive symptomology and multiple co-morbidities may be associated with lower resilience in older carers for several reasons. Caregiving can be a demanding and stressful role, particularly for older adults who may be dealing with their own health challenges. The additional burden of caring for another person can lead to physical, emotional, and cognitive challenges, which can exacerbate the existing illnesses or lead to new ones. Moreover, carers already struggling with their own health may find that when they have multiple co-morbidities, it can be harmful to their physical and emotional health, particularly if they require different management strategies from the care recipient (51). The association of low mood with non-resilience has been shown in several studies, and it has been suggested that carers with higher depressive symptoms may have less capacity to cope with the additional demands of caregiving (39). Research also suggests that an inability to quickly recover from adverse events may increase the risk of vulnerability to mood and anxiety disorders (52).

We found that carers who had attained higher education were more likely to belong to the non-resilient trajectory. This finding conflicts with previous research indicating that higher education is associated with lower depression (53), higher quality of life (54), and lower carer burden (55) in carers of individuals living with dementia. However, it aligns with other research that found that higher-educated spousal carers were more likely to have physical burdens with caregiving activities and were less likely to experience gains from caring (defined as positive experiences through learning, satisfaction, and growing closeness to their partner) (51) and to have lower resilience (56). Additionally, a study in Germany found that while carers with higher education had less physical burden, they had a higher perceived psychological burden, which may be due to a perceived loss of self-fulfillment and autonomy (57). These variations in responses to caregiving may be attributed in part to differing expectations of the carers, particularly differences between those who view their caring role as an obligation rather than a personal choice. For example, in our previous study on grandparental childcare, we found that highly educated grandparents who chose to be heavily involved in caring for grandchildren derived considerable satisfaction from their input, which had a positive impact on their mental health. Conversely, lower-educated grandparents who provided long hours of grandchild care out of economic necessity or obligation experienced poorer mental health outcomes (44).

Finally, we found that caring for an adult child was negatively associated with membership in resilient trajectories. Research investigating the impact of caring for children with mental illness found parents had a lower quality of life and a higher care burden, suggesting that additional supports are necessary for aging parents, including help in accessing available services (58). Parents caring for children with an intellectual disability also have worse mental health (59, 60), and this was greater for those with low emotional social support (60).

### 4.1. Limitations

This study has some limitations. First, as this is an observational cohort, it is not possible to determine causal inference; however, the longitudinal nature and the analysis methodology identifying trajectories of change over time centered around the commencement of the event have added additional evidence about resilience in carers. It should also be noted that the initiation of caregiving may not be the underlying cause of the patterns in the trajectories of SWL. However, the relatively linear patterns of SWL trajectories in the age and sex matched controls who have not initiated caregiving support our hypothesis that these trajectories are associated with caregiving. We selected one wellbeing outcome, satisfaction with life, and modeled change over time to identify resilient trajectories rather than measuring a resilience scale. We adjusted for baseline depressive symptoms to account for the complex relationship between depression, resilience, and life satisfaction; however, there may still be some residual confounding in the potentially bi-directional relationship between resilience and depression. Due to the small sample sizes in some groups, the confidence limits around some estimates are large. However, even with this limitation, indicators were consistent with previous research and the broader literature supporting the important role of social engagement and inclusion for caregivers. Nevertheless, this study has offered some evidence to support potential explanations for the determinants of resilience in carers. Finally, we do not know the exact nature of the disabilities of the care recipients, including adult children. However, carers reported that 65% of adult children cared for had a long-term physical or mental health or disability.

## 5. Conclusion

While much of the focus of research on caregiving has been on negative outcomes associated with caregiving, our study shows that the majority of carers exhibit resilient trajectories in life satisfaction when examined around the timing of becoming a carer. Within the context of aging, resilience is increasingly viewed as an important concept (39, 40, 61). For older adults, resilience encompasses the ability to recover or adapt from a disruption or an event, which includes the emotional and psychological impact of a loved one becoming ill and requiring care. Examining resilience can help identify potential levers to support carers by identifying protective factors that are amenable to intervention at a systems or community level. To date, interventions aimed at reducing carer burden and enhancing their resilience have been diverse and include approaches like meditation, participation in spiritual groups, using technology programs with online options for carers and online health communities (62, 63). However, a recent review of interventions for carers found that they are largely limited to those providing care for individuals living with dementia, and more consideration should be given in the development of interventions to the social determinants of both the caregiver and the care recipient in other circumstances (64). It has been proposed that interventions focused on promoting resilience, purpose-in-life and social integration may be more effective in improving mental health outcomes for carers than interventions specifically for mental health (65). Given the interrelationship between wellbeing and mental health, interventions that support resilience in wellbeing would have similarly positive impacts on the overall mental health of carers. To make the best use of limited formal care resources, health providers should use the indicators identified in this study to screen for those most at risk of poor wellbeing among carers and prioritize these individuals, as well as the recipients in their care, for formal support and assistance. Our study confirms the importance of providing ongoing support and resources for carers to help mitigate the negative effects of caregiving. Interventions that focus on enabling them to identify and strengthen their supportive family and friend networks and provide formal support to enable them to maintain these networks will promote resilience over time.

## Data availability statement

Publicly available datasets were analyzed in this study. This data can be found here: Irish Social Science Data Archive (ISSDA) at University College Dublin, http://www.ucd.ie/issda/data/tilda/; Interuniversity Consortium for Political and Social Research (ICPSR) at the University of Michigan, http://www.icpsr.umich.edu/icpsrweb/NACDA/studies/34315.

## Ethics statement

The studies involving human participants were reviewed and approved by Research Ethics Committee of the Faculty of Health Sciences, Trinity College Dublin. The patients/participants provided their written informed consent to participate in this study.

## Author contributions

CM, MW, and RK contributed to the conception and design of the study, contributed to the interpretation of the findings, manuscript revision, and approval of the final version. CM performed the statistical analysis and drafted the manuscript. All authors contributed to the article and approved the submitted version.
