# Supplementary material for: An investigation of what protective individual- and community-level factors are associated with life satisfaction in middle-aged and older family carers in Ireland
Source: Front Public Health. 2023 Aug 10;11:1207523. doi: 10.3389/fpubh.2023.1207523 (PMC10457003; doi:10.3389/fpubh.2023.1207523)
Supplement: Supplementary file 1 [file Image_1.pdf]

Supplementary Figure 1 Latent growth trajectories (with 95% confidence intervals) of satisfaction with life by time for non carers: Class 1= Stable - High, Class 2= Stable - Low

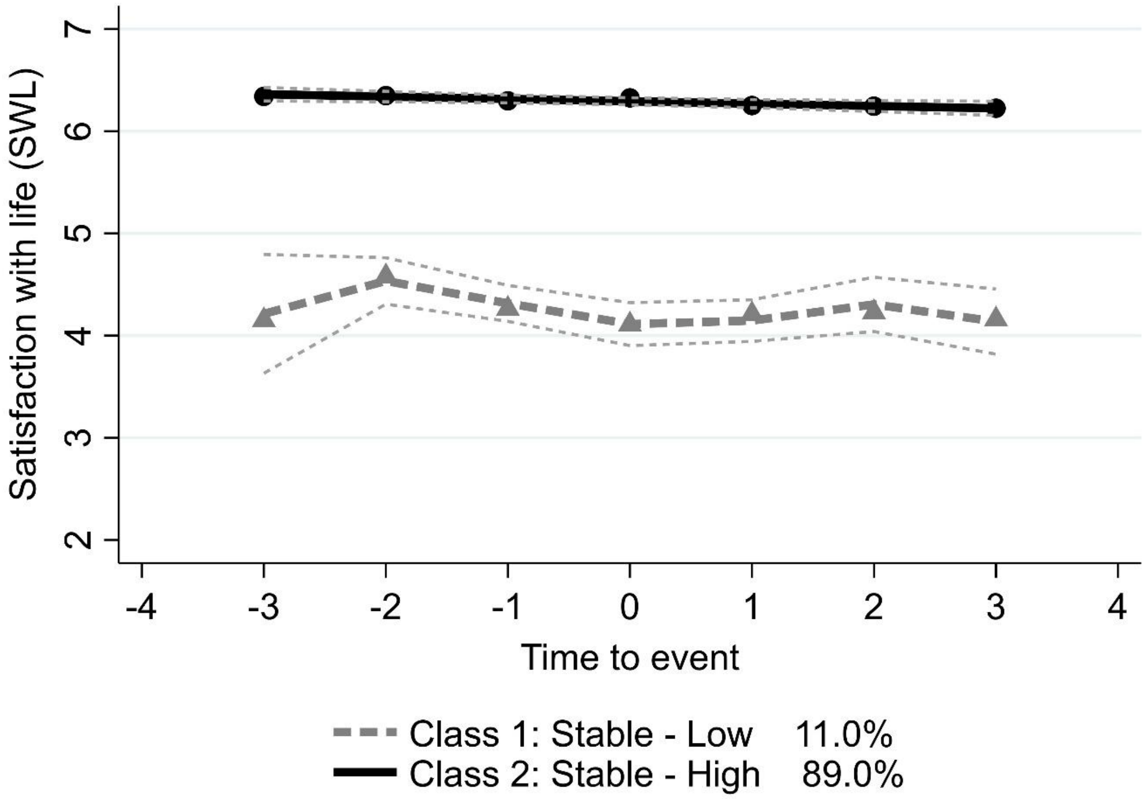

Note: t_0 is nominal time matched to carer, t_{-1} is the interview prior to t_0 (-2 years) and t_{+1} is the interview after t_0 (+2 years)
