# Supplementary material for: An investigation of what protective individual- and community-level factors are associated with life satisfaction in middle-aged and older family carers in Ireland
Source: Front Public Health. 2023 Aug 10;11:1207523. doi: 10.3389/fpubh.2023.1207523 (PMC10457003; doi:10.3389/fpubh.2023.1207523)
Supplement: Supplementary file 2 [file Table_1.DOCX]

Supplementary Table 1 Latent class growth mixture model fit indices

| Class | Count | Average posterior probability | Probability (based on posterior probabilities) | Odds of correct classification (based on weighted posterior probabilities |
| --- | --- | --- | --- | --- |
| Nonrecovery | 42 | 0.92 | 0.056 | 200.7 |
| Resilient-Recovery | 83 | 0.88 | 0.122 | 51.2 |
| Resilient-Stable | 606 | 0.98 | 0.822 | 8.7 |
| Entropy | 0.91 |  |  |  |
| BIC | -5355.57 |  |  |  |
